# Bacteriological and Physicochemical Quality of Drinking Water and Hygiene-Sanitation Practices of the Consumers in Bahir Dar City, Ethiopia

**DOI:** 10.4314/ejhs.v21i1.69040

**Published:** 2011-03

**Authors:** Milkiyas Tabor, Mulugeta Kibret, Bayeh Abera

**Affiliations:** 1 Amhara Region Technical and Vocational Bureau, P.O. Box 2382 Bahir Dar, Ethiopia; 2 Department of Biology, Science College, Bahir Dar University, Bahir Dar, Ethiopia; 3 Department of Microbiology, Parasitology and Immunology, College of Medicine and Health Sciences Bahir Dar University, Bahir Dar, Ethiopia

**Keywords:** Bacteriology, physicochemical, tap water, household, Bahir Dar

## Abstract

**Background:**

Lack of safe drinking water, basic sanitation, and hygienic practices are associated with high morbidity and mortality from excreta related diseases. The aims of this study were to determine the bacteriological and physico-chemical quality of drinking water and investigate the hygiene and sanitation practices of the consumers in Bahir Dar City, Ethiopia.

**Methods:**

A cross sectional prospective study was conducted in Bahir Dar City from October–December, 2009. Water samples were collected from 35 private taps and 35 household water containers for bacteriological analysis. The turbidity, pH, temperature and turbidity were measured immediately after collection. Finally, the hygiene-sanitation practices of the consumers were surveyed using interview.

**Results:**

Twenty seven (77.1%) of the household water samples had high total coliforms counts. Twenty (57.1%) household water samples and 9 (25.7%) of the tap water samples had no residual free chlorine. Sixteen (45.7%) household water samples had very high risk score to thermotolerant coliforms. Eight (22.9%) tap water samples had low risk score for total coliforms whereas 21(60%) tap water had very low risk score for thermotolerant coliforms. Twelve (34.3%) of the consumers collect water without contact with their hand and 9(25.7%) wash their hands with soap after visiting toilet.

**Conclusion:**

Water supplies at tap and household water containers were contaminated with bacteria. Poor sanitation, low level of hygiene, uncontrolled treatment parameters are the causes for contamination. Control of physico-chemical parameters and promoting good hygiene and sanitation are recommended.

## Introduction

Diseases caused by contaminated water consumption and poor hygiene practices are the leading causes of death among children worldwide (1). Lack of safe drinking water, absence of basic sanitation and hygienic practices are associated with high morbidity and mortality from excreta related diseases (2). Water may be contaminated with pathogens at the source but contamination may also occur during distribution, transportation, or handling in households or other working places (3). If raw water is used without treatment, it presents a sanitary risk (4). Inadequate protection of water collection and storage containers and unhygienic conditions contribute to contamination at home (5).

The provision of water, sanitation and good hygiene services is vital for the protection and development of human resources (6). Globally, 1.1 billion people rely on unsafe drinking water sources from lakes, rivers and open wells. The majority of these are in Asia (20%) and sub-Saharan Africa (42%). Furthermore, 2.4 billion people lack adequate sanitation worldwide (7).

Ethiopia is one of the countries in the world with the worst of all water quality problems. It has the lowest water supply and sanitation coverage in Sub-Saharan countries with only 42% and 28% for water supply and sanitation, respectively (8). Most of the population of Ethiopia does not have access to safe and reliable sanitation facilities. Still further, most of its population does not have access to safe and reliable sanitation facilities. On top of these, majority of the households do not have sufficient understanding of hygienic practices regarding food, water and personal hygiene. As a result, over 75 % of the health problems in Ethiopia are due to communicable diseases attributed to unsafe and inadequate water supply, and unhygienic waste management, particularly human excreta (9).

There are a number of pollution sources that continuously deteriorate the bacteriological quality of surface and groundwater in Bahir Dar City. About 60 % of the population use pit latrines (Bahir Dar City Water Supply and Sewerage Service Annual report, Bahir Dar, 2007). The majorities of the pit latrines are often badly constructed improperly maintained and frequently overflow. The liquid waste that is generated by most households in the city either enters the dry pits and septic tanks that are commonly found close to most shelters or simply finds its way to the city's open ditches and swamps (Bahir Dar City Municipality Annual Report, Bahir Dar, 2007). Analysis of drinking water from source to yard connection has been done in Bahir Dar City (10). However, no study has been done on the bacteriological quality of water from the tap to the household and hygiene and sanitation practices of the consumers. The aim of this study was therefore to analyze the bacteriological and physicochemical quality of drinking water at the tap and household and assess the hygiene-sanitation practices of the consumers in Bahir Dar City.

## Materials and Methods

A cross sectional prospective study was conducted in Bahir Dar City from October to December, 2009. Thirty five private taps and 35 household water containers were randomly selected from 11 kebeles. The selection of sampling points and frequency of sampling was determined according the guidelines of WHO (11). Bacteriological and physicochemical quality of water at tap and household water containers were analyzed in three rounds. The hygiene and sanitation practices of the consumers were also assessed.

For bacteriological analysis, 250 ml of water sample was collected between 8:30 and 10:30 am with sterile glass bottle and transported to the laboratory in a cold box. The number of total coliforms and thermotolerant coliforms was determined with the membrane filtration methods using Lauryl Sulfate-Broth (Blulux laboratories Ltd., India) medium (9, 12). For the determination of total coliforms and thermotolerantcoliforms, incubation was carried out at 37°C and 44°C, respectively.

The turbidity and pH of each sample was determined using HI 93703 Microprocessor turbidity Meter (Portugal) and a pH meter CE 370 (EU), respectively, within one hour of collection. The temperature of each sample was determined immediately after collection with a digital thermometer (Multi Thermometer ST-9269, EUROLAB). Free chlorine residual, for each chlorinated sample was determined at the sampling site with a Lovibond 1000 Comparator system (France) using a DPD n°1 chlorine tablet.

Furthermore, consumers' hygiene-sanitation practices were assessed through interview. The interview questions and sanitary inspection forms were adapted from WHO and assessment of the conditions of household water containers was obtained through observation checklist (11). The number of coliforms and hygiene-sanitation inspection rating, risk to health matrix scores were compared with the standard set by WHO (3) and Ethiopian standards (13). Finally, data were recorded, organized and summarized in the form of descriptive statistics using SAS, JMP 501, and SPSS version 12.

Ethical clearance was obtained from the Ethical clearance committee of Bahir Dar University. Data at the households were collected after informed consent was assured from the households. The study objectives were clearly explained to the households and each household was assured that the information provided would be kept confidential.

## Results

In this study, a total of 210 water samples were collected from private taps and household water containers in three rounds. Eight (22.9%), 8(22.9%) and 19(54.2%) tap water samples had total coliform counts ranging from 1.01–9.99, 10–100 and 0 CFU/100 ml, respectively. Four (11.4%), 9(25.7%), 21(60%) samples had 10–100, 1.01–9.99, 0 CFU/100ml thermotolerant coliform counts, respectively. Analysis of household water samples revealed that 19(54.2%) and 12(34.2%) had total coliform count from 10–100 and 1.01–9.99 CFU/100 ml and no household water sample had total coliform count from 0.01–1.01 CFU/100ml. Four (11.4%) household water samples had total coliform count of 0 CFU/ml. In the case of thermotolerant coliforms of the household, 16(45.7%), 14(40%), 1 (2.8%) had counts ranging from 10–100, 1.01–9.99, and 0.01–1.01 CFU/100ml, respectively (Table 1).

**Table 1:** Comparison of bacteriological results of household and tap water with the WHO recommended values, Bahir Dar, 2009

Recommended level of Parameters			Results		
			Tap water	Household water	P- value
Total coliforms (CFU/100ml)					
	10–100		8(22.9%)	19 (54.2%)	< 0.0001
	1.01–9.99		8(22.9%)	12 (34.2%)	
	0.01–1.0		-	-	
	0		19(54.2%)	4 (11.4%)	
		Total	35(100%)	35 (100%)	

Thermotolerant coliforms (CFU/100ml)					
	10–100		4(11.4%)	16 (45.7%)	
	1.01–9.99		9(25.7%)	14 (40%)	< 0.0001
	0.01–1.0		1(2.9%)	1 (2.8%)	
	0		21(60%)	4 (11.4%)	
		Total	35(100%)	35 (100%)	

Out of 35 tap water samples, 11 (31.4%) had temperatures in the range of 15–20°C and 24(68.6%) had temperatures above 20°C. Regarding the residual free chlorine, 11 (31.4%), 15(42.9 %) and 9(25.7%) samples had 2-0.5 mg/l, >0.5mg/l and 0 mg/l, respectively (Table 2). Of 35 household water samples, 24 (68.6%) had temperatures above 20°C with average temperature in the range 17.6–23.4–C. Twenty (57%) of the household water samples had no residual free chlorine. Majority (80–91%) of the samples had pH values in the range 6.5–8.0. There was no difference in the turbidity of water samples in household and tap water samples (Table 2).

**Table 2:** Comparison of physico-chemical results of house hold and tap water sample with the WHO recommended values, Bahir Dar, 2009.

			Results		
Recommended level of Parameters			Tap water	Household water	P- value
Temperature (°C)					
	>20		24(68.6%)	24 (68.6%)	< 0.0001
	15.01–20		11(31.4%)	11 (31.4%)	
	<15		_	_	
		Total	35(100%)	35 (100%)	

Residual Free Chlorine(mg/l)					
	>0.5		6(17.2%)	1 (2.9%)	< 0.0004
	0.2–0.5		11(31.4%)	9 (25.7%)	
	0.1–1.99		9(25.7%)	5 (14.3%)	
	0		9(25.7%)	20 (57.1%)	
		Total	35(100%)	35 (100%)	

pH					
	>8		3(8.6%)	7 (20%)	<0.0001
	6.5–8.0		32(91.4%)	28 (80%)	
		Total	35(100%)	35(100%)	

Turbidity(NTU)					
	>5		1 (2.9%)	1 (2.9%)	0.0931
	2–5		1 (2.9%)	1 (2.9%)	
	0.1–1.99		2 (5.7%)	2 (5.7%)	
	0		31 (88.5%)	31 (88.5%)	
		Total	35 (100%)	35 (100%)	

One way analysis of variance showed that there was statistically significant differences between the tap water and household water containers (p < 0.05) for all the parameters except turbidity (Table 1 and Table 2). Statistically significant differences were found between tap water and household water containers in the mean thermotolerant coliform counts, total coliform counts and in residual free chlorine concentration (Table 3).

**Table 3:** Mean differences of Microbiological and physicochemical parameters at the tap water (n=35) and household water containers (n=35).

Factors	TC		TTC		RFC		Temperature		Turbidity		pH	

Tap and household	Household	Tap	Household	Tap	Household	Tap	Household	Tap	Household	Tap	Household	Tap
Mean difference	20.84	5.61	14.68	3.68	0.14	0.27	20.59	20.61	0.34	0.00	7.76	7.57
Std. Error	1.61	1.61	1.23	1.23	0.02	0.19	0.15	0.15	0.08	0.08	0.03	0.03
Sig.	20.84a	5.61b	14.68a	3.68b	0.14a	0.27b	20.59a	20.61a	0.34a	0.00b	7.76a	7.57b

Key: TC = Total coliforms, TC = Thermotolerant coliforms; RFC= Residual Free chlorine Means followed with the same letter(s) are not significantly different from each other at P= 0.05

In case of risk classification, 54.2% of tap water samples had medium risk score, 8(22.9%) had high risk score and 8(22.9%) samples had low risk score for total coliforms. However, for thermotolerant coliforms, 21(60%) tap water samples had very low risk score and 4 (11.4%) samples had high risk score (Table 4). Using total coliform count as a microbiological indicator to determine the overall risk to health status, 12(34.3%) household water samples had high risk score and 19(54.3%) household water samples had a very high risk score. Using thermotolerant coliform count as a microbiological indicator, 16(45.7%) household water samples had very high risk and 4(11.4%) household water samples had low risk score. The sanitary risk score to health matrix of the household water samples were very high (Table 4).

**Table 4:** Risk-to Health matrix of tap water and household containers Bahir Dar, 2009.

	Total coliforms (CFU /100ml)					Thermotolerant coliforms (CFU /100ml)				

SI score	0	0.01–1	1.01–10	10–100	>100	0	0.01–1	1.01–9.99	10–100	>100
Tap water										

1–2	0	0	0	0	0	0	1	0	0	0
3–5	0	0	0	0	0	0	0	0	4	0
6–8	0	8	0	8	0	0	0	9	0	0
9–12	0	0	19	0	0	21	0	0	0	0
Household container										

0–2	0	0	0	0	0	0	0	1	0	0
3–6	0	0	4	0	0	0	4	0	0	0
7–9	0	0	0	12	19	0	0	0	14	16

Key: SI= Sanitary inspection score, 0 cfu/100ml = Very low risk; 0.01–1cfu/100ml = Low risk; 1.01–10cfu/100ml = intermediate risk; 10–100cfu/100ml = High risk; >100cfu/100ml = Very high risk.

The results of sanitation and hygiene practices of the consumers at the households are shown in Table 5. According to the consumers' responses, 23 (65.7%) collect water without contact with their hands, 18(51.4%) collect water with covered containers and 21(60%) have separate water containers for storing drinking water in the house. Twenty three (65.7%) reported that they wash water collecting containers every day. Thirty one (88.5%) have no latrines in their house and 9(25.7%) replied that they wash their hands with soap after visiting toilet (Table 5). One way analysis of variance showed that there were no statistically significance association between the measured variables and number of indicator bacteria.

**Table 5:** The association between the number of indicator bacteria and sanitation and hygiene practices of the consumers at the household (n=35) Bahir Dar, 2009

Questions asked to the consumers	Responses	

	Yes No(%)	No No(%)
While you are collecting water from the tap, there was contact of the hands to water	12(34.3)	23(65.7)
The water collected from the tap was transported to your house with covered containers?	18(51.4)	17(48.6)
In your house, water for drinking is stored in a separate container from water intended for other purposes	21(60)	14(40)
The drinking water that you take from the storage containers has no contact with your hands.	13(37.1)	22(62.9)
Do you clean your water collection containers every day?	23(65.7)	12(34.3)
Do you have latrine in your house?	4 (11.5)	31(88.5)
After visiting toilet, do you wash your hands with soap?	9(25.7)	26(74.3)

## Discussion

The average count of total coliforms and thermotolerant coliforms were above the recommended value of WHO (4) and Ethiopian Standards (13). In this study, the total coliform and thermotolerant coliform counts were higher in household water samples compared to that of tap water (p= 0.0001). This is in agreement to an intervention study done in Sri Lanka that showed water stored inside the household had often a worse bacteriological quality than water from the source (14). Moreover, other study conducted in Ethiopia indicated that the number of total coliforms in household containers was higher compared to tap water (15). Therefore, compliance is higher for piped water than from household water containers. The results of this study are in agreement with studies conducted in South Africa and Zimbabwe which reported that compliance is significantly higher for tap water (85.4%) than from household water containers (43.6%) (16).

In areas where there is little risk of a waterborne outbreak, residual free chlorine of 0.2 to 0.5 mg/l at all points in the supply is recommended (15). Therefore, when water leaves the treatment plant residual free chlorine of about 1 mg/l is needed for health reasons and it is recommended that such level is maintained at points of consumption (16). In this study, the concentration of residual free chlorine in most water samples were below the recommended limit of WHO (0.2–0.5 mg/l), which indicates the inefficiency of disinfection in the distribution system. This is supported by a case study conducted in rural areas of South Africa (17). General system failures, inefficiency in disinfection, poor maintenance are some of factors that affect the quality of water in Ethiopia (15). The presence of bacteria in water pipes could be attributed to cross-contamination between the municipal water supply and sewer, due to leaky pipes and lack of water pressure (12). According to WHO report re-growth of thermotolerant coliforms in the distribution system are unlikely unless sufficient bacterial nutrients are present or the water temperature is above 15°C, and there is no residual free chlorine (4). Such warm conditions can favor the re-growth of organisms like thermotolerant coliforms in the distribution systems (18). Similar study in Italy showed that the survival curves of *Aeromones* spp. decline rapidly at low temperature (5°C), whereas survival at temperatures greater than 20°C increases (19).

High counts of total coliforms and thermotolerant coliforms at the house hold drinking water indicates that the water has been faecally contaminated. Poor sanitation and poor hygiene in household were the main factors for the contamination water during transportation and after storage at home. A similar drinking water quality assessment study in Ethiopia showed that the majority of household water containers met the recommended limit of WHO and ES and classified as high risk to health classification level. This finding was in agreement to the studies conducted in Ethiopia (15, 20).

In conclusion, the water at the tap and the household were grossly contaminated with bacteria. The tap and household waters were within high health-risk-score. The number of coliforms was above the recommended international and national limits. Poor sanitation, low level of hygiene, and uncontrolled parameters were the causes of the problem. Strict control and appropriate management of the distribution system for prevention of contamination is recommended. Water, sanitation and hygiene education programs should be in place.
